# Recent progress understanding pathophysiology and genesis of brain AVM—a narrative review

**DOI:** 10.1007/s10143-021-01526-0

**Published:** 2021-04-10

**Authors:** Hans-Jakob Steiger

**Affiliations:** 1 Department of Neurosurgery, University Hospital, Düsseldorf, Germany; 2 Department of Neurosurgery, Kantonsspital Aarau, Tellstr. 25, 5001 Aarau, Switzerland

**Keywords:** Brain arteriovenous malformation, Embryology, Genetic mutation, Inflammation, Hemodynamics, 4D MRI, 4D DSA

## Abstract

Considerable progress has been made over the past years to better understand the genetic nature and pathophysiology of brain AVM. For the actual review, a PubMed search was carried out regarding the embryology, inflammation, advanced imaging, and fluid dynamical modeling of brain AVM. Whole-genome sequencing clarified the genetic origin of sporadic and familial AVM to a large degree, although some open questions remain. Advanced MRI and DSA techniques allow for better segmentation of feeding arteries, nidus, and draining veins, as well as the deduction of hemodynamic parameters such as flow and pressure in the individual AVM compartments. Nonetheless, complete modeling of the intranidal flow structure by computed fluid dynamics (CFD) is not possible so far. Substantial progress has been made towards understanding the embryology of brain AVM. In contrast to arterial aneurysms, complete modeling of the intranidal flow and a thorough understanding of the mechanical properties of the AVM nidus are still lacking at the present time.

## Introduction

Arteriovenous malformations of the brain are classically considered tangles of low-resistance channels between arteries and veins. In contrast to arterial aneurysms, relatively little efforts have been made to understand the complexities of hemodynamics and their relation to rupture. Massoud and colleagues elaborated possible flow patterns in analogy to electrical networks [21]. These simulations allowed also appreciating the effect of partial embolization on the flow patterns in the residual AVM. However, these network simulations did not consider flow and pressure patterns within the vascular channels, nor wall tension in the nidal channels, factors that are obviously important determinants for rupture. Thus, there are still a number of secrets around brain AVMs. Batjer and colleagues summarized the state of affairs regarding our understanding of AVM in 2012 like this: “When one inspects a cortically based AVM, one can immediately appreciate the beauty of these lesions. In addition to the striking aesthetics of bright red veins and the tortuous vascularity, the observer has a clear sense of the ominous nature of these lesions” [4].

Since possibilities of invasive measurements during endovascular or surgical therapy are limited, calculations based on models appear mandatory. For the case of arterial aneurysms, the advent of computational fluid dynamics (CFD) has brought a major breakthrough for our understanding. For the case of AVM, on the other hand, this method has so far not resulted in similar progress, since the exact geometry of the AVM compartments, that must been known for CFD calculation, still eludes current imaging methods.

Nonetheless, much progress has been made towards better detail imaging of AVM, including estimation of pressure profiles in the feeding arteries and draining veins. Furthermore, a better understanding of defunct cell signal pathways and somatic mutations allowed for establishing tissue culture models of AV fistulas, resulting in a better understanding of the principles of emergence and growth. The purpose of the current review was to collect the recent scientific results regarding the embryological development of AVM, and regarding advanced imaging and fluid dynamic modeling. The recent literature was surveyed based on a PubMed search on Nov. 20, 2020, with the search terms “brain arteriovenous malformation” and “pathophysiology hemodynamics” (97 hits), “inflammation” (326 hits), “genetic mutation” (131 hits), “hemodynamics MRI” (152 hits), “4D” (44 hits), or “feeding artery pressure” (52 hits).

## Progress understanding the embryology of AVM, germline, and somatic mutations

**Fig. 1:**
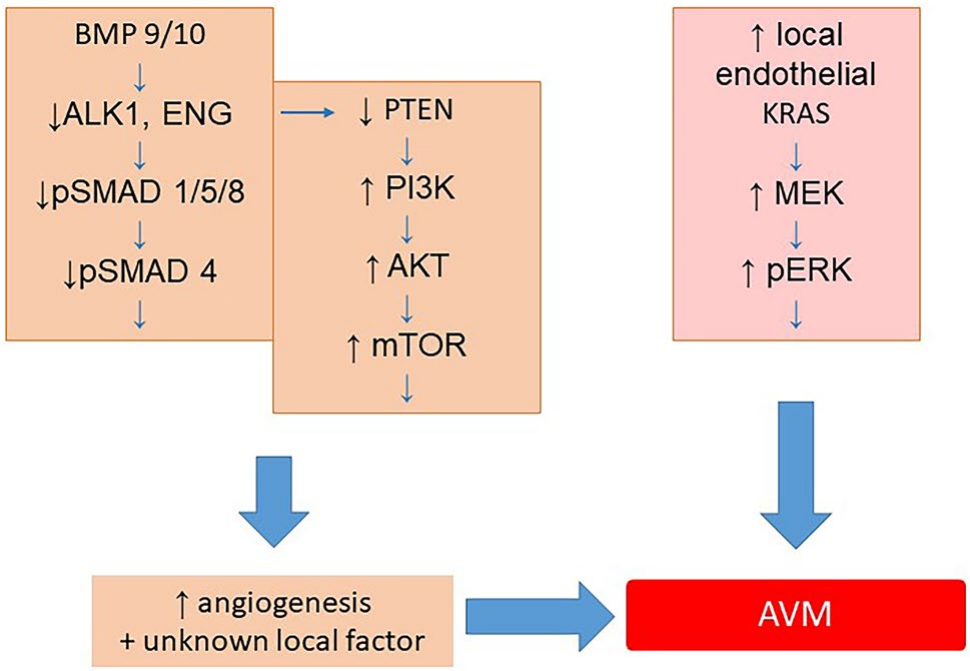
Cell signal pathways involved in development of AVM. Left side: suspected mechanism involved in HHT related AVMs. Normally, BMP9/TGFβ1 regulates angiogenesis through binding to ALK1/ENG to phosphorylate SMAD and increase PTEN activity, which in turn reduces PI3K signaling. HHT mutation of ALK1 or ENG reduces pSMAD and PTEN, resulting in increased PI3K activity or pERK level, causing increased angiogenesis. For the focal development of AVM, an additional local factor must be postulated. Right side: in sporadic AVM cases, somatic activating mutations in genes KRAS, BRAF, or MAP2K1 increase the level of MEK and pERK, leading to AVM development [3]

There is little doubt that AVM are of dysembryological nature. Based on embryological data of normal brain angiogenesis, Mullan and other colleagues believed that AVM develop from a fistula created when during embryogenesis, the developing transverse veins are crossed at right angles by developing longitudinal arteries [34]. The cortical mantle is normally vascularized from the pial surface by an ingrowth of vasogenic cells that canalize and reach the ependymal surface. Later, a cleft occurs in this venous penetration near the ependyma, resulting in a major drainage toward the surface and a minor drainage into a newly formed subependymal venous plexus. Multiple subarachnoid veins connect the pial network with the dural plexus. A failure occurring in the normally compensated surface vein occlusion process was thought by Mullan to initiate the formation of AVM. He postulated in analogy to the development of dural arteriovenous fistulas that thrombosis and abnormal recanalization might play a role for the development of AVM. In contrast to these assumptions, recent evidence suggests that germline and somatic mutations are crucial for AVM development and that aberrant angiogenesis might be the key mechanism (Fig. 1) [3, 53].

### TGF- signaling in familial brain AVM

About 5% of brain AVMs are linked to a genetic disorder, Hereditary Hemorrhagic Telangiectasia (HHT, Rendu-Osler-Weber Syndrome), which is an autosomal dominant vascular disease that affects approximately 1 in 5000 people worldwide [3]. The major clinical feature of HHT is hemorrhage from AVMs in multiple organs, including the brain. Three genes have been identified to cause HHT: *Endoglin* (ENG), *Activin receptor-like kinase 1* (ALK1 or ACVRL1), and *Mothers Against Decapentaplegic Homolog 4* (SMAD4). HHT is classified into HHT1, HHT2, and JP (juvenile polyposis)-HHT, depending on the causative gene mutations. HHT1 (ENG mutations) and HHT2 (ALK1 mutations) cover over 90% of all HHT cases. Although clinical presentations are indistinguishable between HHT1 and HHT2, genotype–phenotype correlation studies have shown that HHT1 has a higher prevalence of AVMs in the brain and lungs, while HHT2 has a higher prevalence of AVMs in the liver and gastrointestinal tract. Brain AVMs are present in 10–16% of patients with HHT [3, 37]. HHT1 patients have a significantly higher brain AVM prevalence (13%) compared with HHT2 patients (< 3%). Most of the HHT-associated AVMs are small (less than 3 cm) and have a Spetzler–Martin grade of 2 or less, whereas in the sporadic AVM population, the mean AVM nidus size is about 3 cm, and the median Spetzler–Martin score is 3. While about 20% of HHT-associated brain AVMs present with rupture, nearly half of the AVMs are asymptomatic. All identified genes associated with HHT are components of signal transduction of TGF-family members; thus, HHT has been considered a disease caused by defects in the signaling of TGF- family members. However, detailed knowledge about the identity of the ligands, receptors, and downstream effectors of ENG-ALK1 signaling pertinent to AVM development are mostly unclear. Recent studies have shown that blockages for both *Bone Morphogenic Protein* BMP9 and BMP10 could induce AVM development in the retinal vasculature, but it is not entirely clear whether both BMP9 and BMP10 are needed for ENG-ALK1 signaling. Nishida analyzed the prevalence of genetic variants in HHT and the correlations to phenotype in 171 patients with HHT and brain AVMs [3]. Twenty-seven percent of patients had a history of cerebral hemorrhage. Multiple brain AVMs were found in 23% of patients. Mutations in ENG, ALK1, and SMAD4 were present in 69%, 17%, and 2%, respectively. To further explore the effect of mutations in ENG, ALK 1, or SMAD 4, Kim and collaborators induced Smad4 deletion in mice at neonatal and adult stages, and looked for hemorrhage and the presence of aberrant arteriovenous connections in various organs [25]. Neonatal Smad4-deleted mice exhibited signs of gastrointestinal bleeding and AVM s in the brain, retina, nose, and intestine. In adult mice, Smad4 deficiency caused gastrointestinal bleeding and AVMs along the gastrointestinal tract and wounded skin. HHT-related phenotypes of Smad4, Alk1, and ENG-deleted mice appeared to be comparable.

Bernabeu and team tried to explain the focal nature of vascular malformations in HHT [6]. They postulated a variety of triggers that may synergize with HHT gene heterozygosity to generate the vascular lesions. Among the postulated second-hits are as follows: mechanical trauma, light, inflammation, vascular injury, angiogenic stimuli, shear stress, modifier genes, and somatic mutations in the wildtype HHT gene allele. Park and collaborators looked into the significance of ALK5- and TGFBR2-independent role of ALK1 in the pathogenesis of HHT type 2 and the role of TGF-beta1 has been considered the most likely ALK1 ligand [40]. The researchers compared the phenotypes of mice and zebrafish in which the ALK1, ALK5, or TGFBR2 gene was conditionally deleted in restricted vascular endothelia. ALK1-conditional deletion resulted in severe vascular malformations mimicking all pathologic features of HHT, yet ALK5- or TGFBR2-conditional deletion in mice, or ALK5 inhibition in zebrafish, did not affect vessel morphogenesis. These data suggested that neither ALK5 nor TGFBR2 is required for ALK1 signaling pertinent to the pathogenesis of HHT and suggest that HHT might not be a TGF-beta subfamily disease. From the same team, Corti et al. studied the interaction between ALK1 and blood flow in the development of arteriovenous malformations [11]. They analyzed AVM development in zebrafish embryos harboring a mutation in ALK1. Their analyses demonstrated that increases in arterial caliber, which stem in part from increased cell number and in part from decreased cell density, precede AVM development, and that AVMs represent enlargement and stabilization of normally transient arteriovenous connections. Whereas initial increases in endothelial cell number are independent of blood flow, later increases, as well as AVMs, are dependent on flow. Furthermore, they could demonstrate that ALK1 expression requires blood flow, and that despite normal levels of shear stress, some flow-responsive genes are dysregulated in ALK1 mutant arterial endothelial cells. Taken together, the results suggest that Alk1 plays a role in transducing hemodynamic forces into a biochemical signal required to limit nascent vessel caliber, and support a novel two-step model for HHT-associated AVM development in which pathological arterial enlargement and consequent altered blood flow precipitate a flow-dependent adaptive response involving retention of normally transient arteriovenous connections, thereby generating AVMs.

A more recently described rare systemic disorder associated with AVM is *Capillary Malformation-Arteriovenous Malformation* (CM-AVM) syndrome [5, 12]. This syndrome is characterized by the presence of multiple small (1–2 cm in diameter) capillary malformations mostly localized on the face and limbs. Some affected individuals also have associated arteriovenous malformations (AVMs) and/or arteriovenous fistulas (AFVs) arising in the skin, muscle, bone, spine, and brain. Symptoms from intracranial AVMs/AVFs appear to occur early in life. Several affected individuals have Parkes-Weber syndrome (multiple micro-AVFs associated with a cutaneous capillary stain and excessive soft-tissue and skeletal growth of an affected limb). The diagnosis of CM-AVM is established by a heterozygous pathogenic variant in *Ephrin-Typ-B-Rezeptor 4* (EPHB4) or *RAS p21 Protein Activator 1* (RASA1) identified by molecular genetic testing. CM-AVM syndrome is inherited in an autosomal dominant manner. For RASA1-CM-AVM syndrome, about 70% of affected individuals have an affected parent; about 30% have a de novo pathogenic variant. For EPHB4-CM-AVM syndrome, some 80% of affected individuals have an affected parent; about 20% have a de novo pathogenic variant. Each child of an individual with CM-AVM syndrome has a 50% chance of inheriting the pathogenic variant. Prenatal and preimplantation genetic testing is possible if the pathogenic variant has been identified in an affected family member.

### RAS-MAPK-ERK signaling in sporadic brain AVM

The *Rat Sarcoma* (RAS)/*Mitogen-Activated Protein Kinase* (MAPK)/*Extracellular-Signal-Regulated Kinase* (ERK) cascade couples signals from cell surface receptors to transcription factors, which regulate gene expression. This pathway regulates several critical cellular functions, including proliferation, growth, survival, and senescence [3]. It has been shown that alteration of the RAS-MAPK pathway triggers the development of tumors. Further studies showed that *Kirsten Rat Sarcoma* (KRAS) mutations are present in colorectal, lung, and biliary tract carcinogenesis, while mutations in *Neuroblastoma RAS* (NRAS) and *Harvey RAS* (HRAS) are frequently present in melanomas and salivary gland tumors, respectively. Interestingly, almost all pancreatic adenocarcinomas harbor a RAS mutation. Furthermore, it has been shown that ERK5 regulates several signaling pathways involved in angiogenesis, whereby it can inhibit the expression of VEGF during the hypoxic response.

Regarding truly sporadic brain AVM, Nikolaev from the University of Geneva, and colleagues examined tissue and blood samples of patients with brain arteriovenous malformations to detect somatic mutations [36]. The researchers identified somatic activating KRAS mutations in AVM tissue samples from 45 of the 72 patients; mutations were not identified in any of the 21 paired blood samples. KRAS mutations were detected in endothelial cell-enriched cultures derived from arteriovenous malformations of the brain. Expression of mutant KRAS in endothelial cells in vitro induced increased ERK activity, increased expression of genes related to angiogenesis and Notch signaling, and enhanced migratory behavior. Inhibition of MAPK-ERK signaling resulted in reversal of these processes. Fish and coworkers showed in mice and zebrafish that endothelial-specific gain of function mutations in KRAS (G12D or G12V) is sufficient to induce brain arteriovenous malformations [13]. Active KRAS signaling was found to lead to altered endothelial cell morphogenesis and increased cell size, ectopic sprouting, expanded vessel lumen diameter, and direct connections between arteries and veins. Furthermore, the researchers demonstrated that these lesions are not associated with altered endothelial growth dynamics or a lack of proper arteriovenous identity but instead seem to feature exuberant angiogenic signaling. Finally, it was demonstrated that KRAS-dependent arteriovenous malformations in zebrafish are refractory to inhibition of the downstream effector PI3K but instead require active MEK (MAPK1) signaling. Hong and coworkers also found a high prevalence of KRAS and *B-Rat Fibro sarcoma* (BRAF) gene somatic mutations in brain and spinal cord arteriovenous malformations [19]. Similarly, Gross and coworkers described somatic mutations in endothelial cells of AVM. Mutations concerned KRAS in the majority and or BRAF in a few [15]. Priemer described a somewhat lower incidence of activating KRAS mutations in their AVM samples [43]. They sought to determine the frequency of KRAS mutations and their association with clinical characteristics. KRAS mutations were found in 6 of 21 cases tested (28.5%). Five mutations were p.G12 V, and one p.G12C. The KRAS-mutant group contained 4 females and 2 males, with an average age of 28 years, compared to 34 years in the non-mutant group (*P* = 0.54). There were no histologic differences between KRAS-mutant and non-mutant cases.

Walcott described a rare somatic mutation of the *Bone Morphogenic Protein* (BMP) pathway in the arteriovenous malformation of a 14-year-old girl who developed a recurrent brain AVM [52]. Whole-exome sequencing of AVM tissue and blood was performed accompanied by in silico modeling, protein expression observation in lesion tissue and zebrafish modeling. A stop-gain mutation (c.C739T:p.R247X) in the gene SMAD family member 9 (SMAD9) was discovered. In the human brain tissue, immunofluorescent staining demonstrated a vascular predominance of SMAD9 at the protein level. Vascular SMAD9 was markedly reduced in AVM peri-nidal blood vessels, which was accompanied by a decrease in phosphorylated SMAD4, a downstream effector protein of the BMP/MEK signaling pathway. Zebrafish modeling (Tg kdrl:eGFP) of the morpholino splice site and translation-blocking knockdown of SMAD9 resulted in abnormal cerebral artery-to-vein connections with morphologic similarities to human AVMs. Hill-Fellberg and coworkers analyzed Notch receptor expression in brain AVM [18]. They found that compared to normal brain vascular tissue, Notch-3 was dramatically increased in brain arteriovenous malformations. Similarly, Notch 4 labelling was also increased in vascular malformations and was confirmed by western blot analysis. Notch 2 was not detectable in any of the human vessels analyzed. Using both immunohistochemistry on microarrays and western blot analysis, they found that Notch-1 expression was detectable in control vessels, and discovered a significant decrease of Notch 1 expression in vascular malformations. They demonstrated that Notch 3 and 4, and not Notch 1, were highly increased in human arteriovenous malformations.

Summarizing these genetic analyses, it can be concluded that sporadic AVM are consequences of local somatic mutations affecting the endothelium. The situation is more complex in the situation of the rare familial cases, mainly in context with HHT. Here, in addition to a germline mutation, local additional factors must be postulated for the emergence of AVMs. These second factors are not well understood at the present time.

## The role of inflammation for development and rupture

Comparable to intracranial aneurysms, inflammatory processes have been suspected for years to have a potential influence for the development and rupture of arteriovenous malformations [26, 30, 31, 33, 55]. The advent of ferumoxytol-enhanced and later gadolinium-enhanced Black Blood MRI (BbMRI) allowed us to visualize contrast leak as a surrogate of inflammation [8, 42]. Petridis and collaborators used the latter method to characterize ruptured and unruptured AVM and AV-fistulas [42]. They found contrast enhancement in 9 of 10 niduses or fistulas respectively, irrespective of rupture status or size. They concluded that BbMRI is a feasible method of identifying inflammation in AVM and that, in contrast to unruptured saccular aneurysms, high-flow malformations are in a permanent stage of inflammation, which does not seem to allow conclusions on their rupture risk at the current stage. This result corroborates well with recent histopathological studies. Wright and coworkers analyzed tissue samples of 85 AVMs with histology and CD45 immunostainings [54]. The histological data was compared with the clinical history of the patients. Inflammation was found in all studied AVMs and did not correlate with rupture. While multiple types of inflammatory cells were present, macrophages were clearly the dominant inflammatory cell type, especially in samples with strong inflammation. Of those AVMs with strong inflammation, only 56% had presented with clinically evident rupture. However, hemosiderin which is a sign of prior hemorrhage was detected in 79% (58/74) of samples with strong inflammation and was associated with it (*P* = 0.003). The authors also concluded that strong inflammation is present in both unruptured and ruptured AVMs.

The finding that microhemorrhages are common in ruptured as well as unruptured AVMs corroborates well with the microscopic observations made by McCormick already more than 50 years ago. He described AVMs histopathologically as follows: “Microscopically, the variable and extremely complex nature of the lesion becomes evident. The vessels may range from relatively well-differentiated arteries and veins to malformed, thick and thin-walled, hyalinized vessels apparently neither artery nor vein. Segmental dilatations of these vessels are often seen. Large, irregular nodules of hyalinized intima and smooth muscle often project into the lumens. A peculiar, amyloid-like material is at times found in the vessel walls. Ossification may also occur, but is uncommon. Degeneration of the parenchyma about and within the malformation is almost constant, and may be the most conspicuous feature in some cases. Hemosiderin pigment is commonly found, indicating at least minor hemorrhage from the malformation” [32]. Hemosiderin may well be associated with inflammation and be the main cause of vessel wall enhancement found with black-blood MRI, as mentioned above [42].

Hauer et al. recently used RNA sequencing to study upregulation of inflammatory cytokines [16]. They used next-generation RNA sequencing to identify differential expression on a transcriptome-wide level comparing tissue samples of 12 AVMs to 16 intracranial control arteries. They found 736 upregulated genes in AVMs and 498 downregulated genes, including genes implicated in extracellular matrix composition, the binary angiopoietin-TIE system, and TGF-β signaling, and inflammation. They concluded that the results point to involvement of inflammatory mediators, loss of cerebrovascular quiescence, and impaired integrity of the vascular wall in the pathophysiology of brain AVMs.

Neyazi and collaborators studied *Carcinoembryonic Antigen-related Cell Adhesion Molecule 1* (CEACAM1), which is associated with infiltrating neutrophils, in tissues samples of 60 brain AVM [35]. The association of CEACAM1 with clinical parameters was analyzed. High levels of CEACAM1-positive cells were associated with AVM rupture, but not with AVM size, preoperative embolization, or seizures. This association with rupture status was significant only in male but not in female patients. Within the ruptured AVM group, patients with a short hemorrhage to surgery time interval had higher levels of CEACAM1 immune infiltration than patients with long interval. This decrease in the levels of CEACAM1 immune infiltration between the short and long interval groups was, however, significant only in female patients. The authors suggest the presence of a sexual dimorphism regarding inflammation in AVM.

Pawlinowska and her team genotyped 180 AVM patients for 5 polymorphisms in 3 inflammatory cytokine genes, and 9 polymorphisms in 5 angiogenesis-related genes [41]. They hoped that identification of genetic polymorphisms associated with AVM hemorrhage would facilitate risk stratification in patients. Patients homozygous for the interleukin 6 (IL6)-174G allele had a greater risk of ICH presentation (OR, 2.62, *P* = 0.003) than IL6-174C carriers. In a multivariate logistic regression model, IL6-174G > C genotype, as well as the known predictors small BAVM size, and exclusively deep venous drainage were independent predictors of ICH presentation [24].

Summarizing the present state regarding the importance of inflammation for rupture of AVM, we can conclude that inflammation in AVM does certainly occur. However, further studies are needed to define the role of inflammatory cytokines in the pathogenesis of AVM hemorrhage, before possible preventive use of anti-inflammatory drugs can be considered [56, 57].

## Understanding blood flow and pressure gradients in AVM

### Advanced MRI

Advanced time-resolved MRI analysis has considerably advanced our understanding of AVM hemodynamics. Phase contrast imaging provides a vector field of the velocity and yields additional hemodynamic information, including volume flow rate and intravascular pressure. Color-coded images can separate feeding arteries, nidus, and draining veins. Theoretically, the images provide local flow velocities. In combination with the measured vascular diameters and lengths, the pressure drop along the feeding arteries and draining veins can be calculated. Ansari and coworkers were among the first to introduce this method. They aimed to determine whether time-resolved (4D) flow MR could provide insights into arteriovenous malformation hemodynamics and the changes in flow observed during staged embolization [1]. They found that velocities in the draining veins increased from Spetzler-Martin grade 1 to Spetzler-Martin grade 3 and above, whereas arterial velocities were similar in all Spetzler-Martin grades. Li and colleagues used the method of time-resolved (4D) flow MRI for follow-up after stereotactic radiosurgery of parenchymal AVM [28, 29]. They found a measurable reduction of AVM flow already after 6 months. MacDonald et al. also published calculations of intravasal flow and pressures in aneurysms and AVM using phase contrast magnetic resonance imaging [30, 31]. They found that patients with aneurysms had reduced flow in vessels distal to the aneurysm, while AVM patients had increased flow in some vessels supplying and draining the nidus. Calculated pressures in aneurysms were highly variable between subjects and location, while in the nidus of the AVMs, pressure trended higher in larger AVMs. Iryo and team evaluated intracranial arteriovenous malformations with 4-D MRI [20]. In high-flow AVMs, the color-coded maps were especially helpful for identifying the feeders and drainers. Chang and collaborators studied fast contrast-enhanced 4D MRA and 4D flow MRI using constrained reconstruction (HYPRFlow) for 21 brain arteriovenous malformations [9]. HYPRFlow was equivalent to 3D-TOF in delineating normal arterial anatomy, arterial feeders, and nidus size and was concordant with DSA for AVM grading and venous drainage. Mean arterial transit time in the AVM hemisphere was 0.5 s, and on the normal contralateral side, 2.5 s. The mean arterial volume flow rate in the M1 segment ipsilateral to the AVM was 4 ± 3 mL/s and in the contralateral M1 segment 2 ± 0.6 mL/s. Raoult and coworkers tried hemodynamic quantification in brain AVMs with time-resolved spin-labeled magnetic resonance angiography [43]. Among the quantitative parameters, time-to-peak and maximum outflow gradient allowed discriminating various intranidal flow patterns with significant differences between feeding arteries and draining veins. With nine AVMs classified into the high rupture risk group (six of them hemorrhagic) and seven into the low rupture risk group, the observed venous-to-arterial time-to-peak ratio was significantly lower in the high rupture risk and hemorrhagic groups. Kumar and colleagues compared computer flow simulation with MRA for evaluating AVM hemodynamics [26, 27, 28]. Mean flow in defined vessels, diameter, and pressure were compared between modeling results and validation measurements using special flow software, showing that modeling results and measurements were matching with a small deviation. Chang and team also used high-resolution 3D radial phase-contrast MR angiography for hemodynamic evaluation in patients with arteriovenous malformations [10]. Ten patients with AVMs were scanned and flow velocity and wall shear stress in vessels feeding the AVMs and normal contralateral vessels were calculated using velocity data from the phase-contrast acquisition. Patients with an asymptomatic presentation or mild symptoms had no significant difference in wall shear in feeding vessels compared with the normal contralateral vessels, whereas patients presenting with hemorrhage, severe headaches or seizures, or focal neurologic deficits had significantly higher wall shear stress in feeding arteries compared with contralateral vessels.

The abovementioned results show that advanced MRA provided some progress regarding analysis of flow through the AVM. The method does, however, not allow a detailed analysis of the highly complex flow patterns in the nidus. Furthermore, calculations of pressure profiles along the feeding arteries and highly sensitive to small errors concerning measured vessel diameters.

### Advanced DSA

**Fig. 2:**
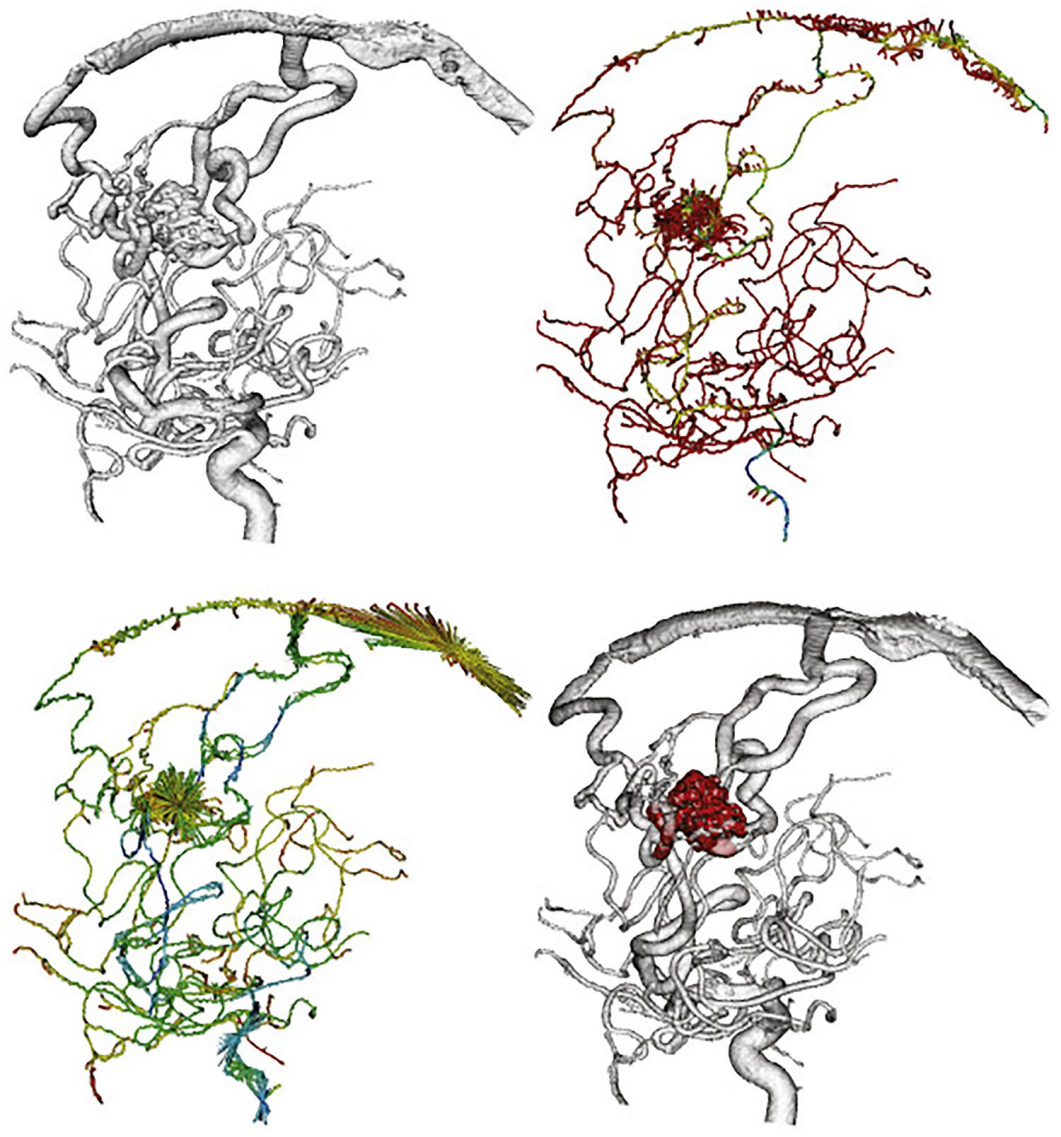
Advanced algorithms for vessel separation in AVM digital subtraction angiography. A skeleton of the 3-D images of the vascular tree is generated in order to separate the segments of the AVM. From Babin et al. with kind permission from Elsevier [2]. Time-resolved MRI sequences allow similar decomposition of vascular segments

Several groups have propagated advanced DSA imaging including visualization of flow fields [7, 23, 39]. This method appears to be consistent with computational fluid dynamics in evaluating inflow direction and recirculation in aneurysms. Moreover, the method promises also new solutions for improving the visibility of flow patterns when contrast motion in DSA is not apparent. Regarding AVM, Babin and colleagues used a graph-based skeletonization method for vessel delineation in arteriovenous malformations with the main purpose of AVM decomposition into veins, arteries and the nidus (see Fig. 2) [2]. Similarly, the teams of Ogard, and also Sandoval-Garcia used advanced angiographic exploration in AVM patients with both 4D DSA and 2D DSA [37, 38, 45, 46]. A good agreement between the techniques was found regarding Spetzler-Martin grade and nidus size, but less for defining the venous drainage. Ruedinger and colleagues worked on the optimization of 4D-DSA [44, 45]. They used two 3D-printed patient-specific models connected to a pulsatile pump and flow system. 4D-DSA acquisitions were performed for the various contrast injection rates. The strongest pulsatility signal occurred with the 2.5 mL/s injections. The largest oscillation amplitudes were found with 2.0- and 2.5-mL/s injections. Geometric accuracy was best preserved with injection rates of > 1.5 mL/s.

Summarizing the results of advanced DSA, similar limitations appear to apply as with time-resolved MRI. The detailed flow patterns in the nidus appear still difficult to grasp in detail.

### Pressure measurements

Fogarty-Mack and colleagues measured feeding artery pressure during embolization in order to examine the distribution of arterial hypotension surrounding arteriovenous malformations [14]. Mean arterial pressures were recorded during superselective cerebral angiography in 96 patients with AVMs, before they underwent liquid polymer embolization. Distal mean arterial pressure at the site of polymer injection amounted to approximately 40 mmHg corresponding to 50% of the systemic arterial pressure. Henkes and colleagues also made pressure measurements in arterial feeders of arteriovenous malformations during endovascular treatment procedures [17]. A total of 148 measurements were performed in 139 patients. Pressure values were correlated with various clinical parameters (i.e., AVM location, size, previous hemorrhage) and pathoanatomical features of the AVM (e.g., nidus structure, number of draining veins). Distal mean feeder pressure values were 55 mmHg on average. Pressure was lower in more distally located AVMs, in larger lesions and in AVMs with multiple drainage veins. Pressure values were significantly higher in patients with previous hemorrhage and in smaller AVMs. Sorimachi and team also studied the hemodynamics of arteriovenous malformations during embolization and measured pressures in 21 feeders in 14 patients through a microcatheter system [48, 49]. Before embolization, the pressures were significantly lower in feeders with branches terminating in the malformation (terminal divided branches) and comparatively low in arteriovenous malformations with rapid blood flow through the malformation. The pressures in feeders with transient branches supplying brain were significantly higher.

**Fig. 3:**
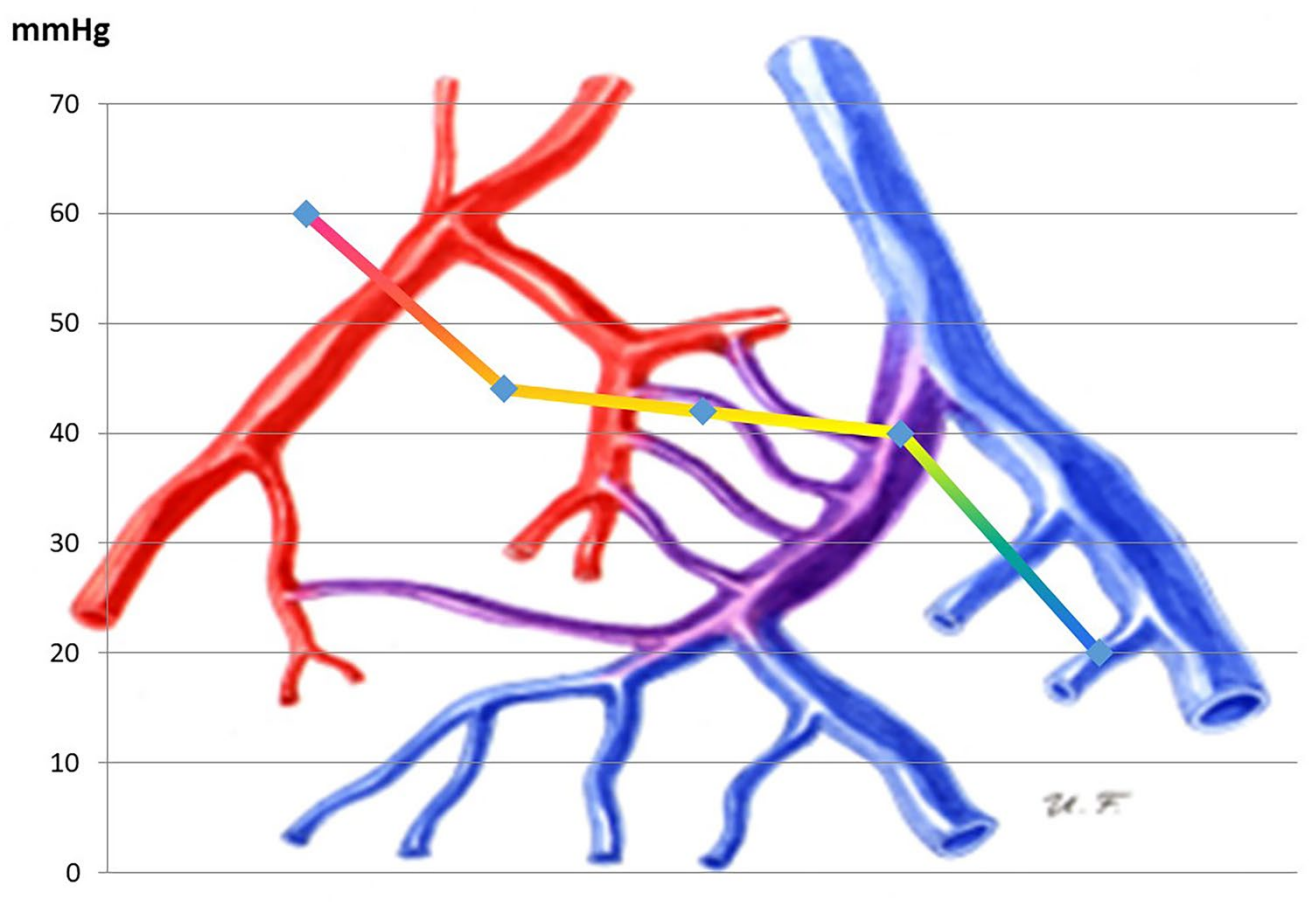
Pressure drop from arterial to venous side, as extrapolated from pressure measurements and CFD simulations. The main drop of arterial pressure takes place along the feeding arteries. Pressure gradient along the nidal vessels appears to be usually quite shallow

Some additional insight regarding AVM hemodynamics might also be gained from less advanced technical methods. We used simple native and contrast CT to estimate the cumulative vessel diameter at the nidal and draining vein level. In 10 AVMs measured, we found an average ratio of total nidal vascular diameter to draining vein cross-area of 5.8 (Steiger, unpublished). This suggests, on the other hand, that net flow velocity in the nidus is almost 6 times slower than in the draining veins, and that pressure drop along the nidus is relatively small (Fig. 3).

## Pathophysiologcal considerations

### Mechanical concepts

Pathophysiologically, AVMs have been commonly modeled as tangles or networks of small channels [21, 22, 38]. For a more precise understanding, the interaction between the adjacent channels and the properties of the interstitium should also be taken into consideration. Unfortunately, no data is currently available regarding interstitial pressure and tissue strength. We depend therefore on theoretical considerations, which will be detailed in the following.

A certain stability is a mandatory precondition for an AVM structure to persist to adulthood. This precondition still leaves a number of structures as possible AVMs. In the end, there are probably also various possible angio-architectures, a fact that is also reflected by the histopathological variants, e.g., direct arteriovenous fistula, compact nidus, racematous plexiform nidus, capillary type of nidus. Generally speaking, the intranidal pressure is contained by (1) the vessel walls and (2) the gliotic interstitial tissue. If nidal vessels are not contained by the gliotic interstitial tissue, the wall tension and risk of rupture are likely to be governed by Laplace’s law (wall tension = pressure × radius), suggesting that rupture would preferentially occur close to the arterial side, with higher intravasal pressure. On the other hand, if the channels of the nidus are glued together by the gliotic interstitial tissue, the dynamics are somewhat different [50, 51]. Rupture could obviously occur only at the surface of the nidus, preferentially also on the arterial side. This scenario appears to correspond to what we know from reality.

If the nidal channels are glued together by the interstitial tissue, this has also some other consequences. First, the interstitial pressure would correspond to the intravasal pressure. Second, the pressure in adjacent channels must be almost identical in order to prevent compression of one lower pressure channel by the neighboring higher-pressure channel. This concept would render true tangles or knots inside the nidus unlikely and therefore suggest a clear polarity and alignment in the nidus of an AVM. This picture appears also to be in agreement with the usual angiographic appearance.

Theoretically, a spherical structure of an AVM can be imagined, with the feeding arteries entering to the center and the draining veins draining from the surface of the sphere. Such a nidus could hardly bleed, since the nidal pressure would approach normal venous pressure at the surface. Such a structure is not known to occur in reality. The reason is probably that draining veins cannot encompass the entire nidus. Draining veins appear rather to develop from a venous pole in a fingerlike fashion toward an arterial pole, or in other words, the venous side develops as a fractal tree-like branching structure with all of the branches accepting direct AV-Shunts from an arterial network developing between the tree branches.

### Flow instability and turbulent flow in AVM

Flow instabilities and even frank turbulence are well known from intracranial aneurysms, although the significance for growth and rupture remains unclear [47, 49]. In dural arteriovenous fistulas, the existence of turbulent flow is the obvious origin of pulsatile tinnitus, which is often the presenting complaint. In AVM, on the other hand, little research has been done regarding the occurrence and potential impact of flow instabilities. Patients harboring parenchymal AVM rarely complain of pulsatile tinnitus. Only Sekhar and colleagues noted, with the help of an electronic stethoscope, vascular bruits also arising from brain AVMs [47]. Flow instabilities in aneurysms occur, as in other flow scenarios, at the sites of flow deceleration [48, 49]. Referring to our abovementioned results suggesting a substantial flow deceleration at the transition from terminal feeders to the nidus, we would assume some flow instability in the AVM nidus.

## Conclusions

Substantial advancements have been made during recent years to understand the embryological origin of brain AVM and the somatic and germline genetic mutations involved. Advances of imaging modalities allowed also better understanding the blood flow in feeding arteries and draining veins. Nonetheless, in contrast to arterial aneurysms, complete modeling of the intranidal flow by computational fluid dynamics (CFD) is not possible so far and a thorough understanding of the mechanical properties of the AVM nidus is still lacking for the time being. The relation of inflammation and flow pattern and to the risk of hemorrhage remains incompletely understood.

## References

[1] Ansari SA, Schnell S, Carroll T, Vakil P, Hurley MC, Wu C, Carr J, Bendok BR, Batjer H, Markl M (2013). Intracranial 4D flow MRI: toward individualized assessment of arteriovenous malformation hemodynamics and treatment-induced changes. AJNR Am J Neuroradiol.

[2] Babin D, Pižurica A, Velicki L, Matić V, Galić I, Leventić H, Zlokolica V, Philips W (2017). Skeletonization method for vessel delineation of arteriovenous malformation. Comput Biol Med.

[3] Barbosa Do Prado L, Han C, Oh SP, Su H, Han C, Oh SP, Su H (2019). Recent advances in basic research for brain arteriovenous malformation. Int J Mol Sci.

[4] Batjer HH, Aoun Sala G, Rahme Rudy J, Bendok Bernard R (2012). Red cerebral veins. The science, the art and the craft. Neurosurgery.

[5] Bayrak-Toydemir P, Stevenson DA (2011) Capillary malformation-arteriovenous malformation syndrome. In: Adam MP, Ardinger HH, Pagon RA, Wallace SE, Bean LJH, Stephens K, Amemiya A, editors. GeneReviews® [Internet]. Seattle: University of Washington, Seattle; 1993–2020 21348050

[6] Bernabeu C, Bayrak-Toydemir P, McDonald J, Letarte M (2020). Potential second-hits in hereditary hemorrhagic telangiectasia. J Clin Med.

[7] Brina O, Ouared R, Bonnefous O, van Nijnatten F, Bouillot P, Bijlenga P, Schaller K, Lovblad KO, Grünhagen T, Ruijters D, Pereira VM (2014). Intra-aneurysmal flow patterns: illustrative comparison among digital subtraction angiography, optical flow, and computational fluid dynamics. AJNR Am J Neuroradiol.

[8] Chalouhi N, Jabbour P, Magnotta V, Hasan D (2014). Molecular imaging of cerebrovascular lesions. Transl Stroke Res.

[9] Chang W, Wu Y, Johnson K, Loecher M, Wieben O, Edjlali M, Oppenheim C, Roca P, Haild J, Aagaard-Kienitz B, Niemann D, Mistretta C, Turski P (2015). Fast contrast-enhanced 4D MRA and 4D flow MRI using constrained reconstruction (HYPRFlow): potential applications for brain arteriovenous malformations. AJNR Am J Neuroradiol.

[10] Chang W, Loecher MW, Wu Y, Niemann DB, Ciske B, Aagaard-Kienitz B, Kecskemeti S, Johnson KM, Wieben O, Mistretta C, Turski P (2012). Hemodynamic changes in patients with arteriovenous malformations assessed using high-resolution 3D radial phase-contrast MR angiography. AJNR Am J Neuroradiol.

[11] Corti P, Young S, Chen CY, Patrick MJ, Rochon ER, Pekkan K, Roman BL (2011). Interaction between alk1 and blood flow in the development of arteriovenous malformations. Development.

[12] Eerola I, Boon LM, Mulliken JB, Burrows PE, Dompmartin A, Watanabe S, Vanwijck R, Vikkula M (2003). Capillary malformation-arteriovenous malformation, a new clinical and genetic disorder caused by RASA1 mutations. Am J Hum Genet.

[13] Fish JE, Flores Suarez CP, Boudreau E, Herman AM, Gutierrez MC, Gustafson D, DiStefano PV, Cui M, Chen Z, De Ruiz KB, Schexnayder TS, Ward CS, Radovanovic I, Wythe JD (2020). Somatic gain of KRAS function in the endothelium is sufficient to cause vascular malformations that require MEK but not PI3K signaling. Circ Res.

[14] Fogarty-Mack P, Pile-Spellman J, Hacein-Bey L, Osipov A, DeMeritt J, Jackson EC, Young WL (1996). The effect of arteriovenous malformations on the distribution of intracerebral arterial pressures. AJNR Am J Neuroradiol.

[15] Goss JA, Huang AY, Smith E, Konczyk DJ, Smits PJ, Sudduth CL, Stapleton C, Patel A, Alexandrescu S, Warman ML, Greene AK (2019). Somatic mutations in intracranial arteriovenous malformations. PLoS ONE.

[16] Hauer AJ, Kleinloog R, Giuliani F, Rinkel GJE, de Kort GA, Berkelbach van der Sprenkel JW, van der Zwan A, Gosselaar PH, van Rijen PC, de Boer-Bergsma JJ, Deelen P, Swertz MA, De Muynck L, Van Damme P, Veldink JH, Ruigrok YM, Klijn CJM (2020). RNA-sequencing highlights inflammation and impaired integrity of the vascular wall in brain arteriovenous malformations. Stroke.

[17] Henkes H, Gotwald TF, Brew S, Miloslavski E, Kämmerer F, Kühne D (2006). Intravascular pressure measurements in feeding pedicles of brain arteriovenous malformations. Neuroradiology.

[18] Hill-Felberg S, Wu HH, Toms SA, Dehdashti AR (2015). Notch receptor expression in human brain arteriovenous malformations. J Cell Mol Med.

[19] Hong T, Yan Y, Li J, Radovanovic I, Ma X, Shao YW, Yu J, Ma Y, Zhang P, Ling F, Huang S, Zhang H, Wang Y (2019). High prevalence of KRAS/BRAF somatic mutations in brain and spinal cord arteriovenous malformations. Brain.

[20] Iryo Y, Hirai T, Nakamura M, Kawano T, Kaku Y, Ohmori Y, Kai Y, Azuma M, Nishimura S, Shigematsu Y, Kitajima M, Yamashita Y (2016). Evaluation of intracranial arteriovenous malformations with four-dimensional arterial-spin labeling-based 3-T magnetic resonance angiography. J Comput Assist Tomogr.

[21] Jain MS, Do HM, Massoud TF (2019). Computational network modeling of intranidal hemodynamic compartmentalization in a theoretical three-dimensional brain arteriovenous malformation. Front Physiol.

[22] Jayalalitha G, Deviha V, Uthayakumar R (2008). Fractal model for blood flow in cardiovascular system. Comput Biol Med.

[23] Kato N, Yuki I, Hataoka S, Dahmani C, Otani K, Abe Y, Kakizaki S, Nagayama G, Maruyama F, Ikemura A, Kan I, Kodama T, Ishibashi T, Murayam Y (2020). 4D digital subtraction angiography for the temporal flow visualization of intracranial aneurysms and vascular malformations. J Stroke Cerebrovasc Dis.

[24] Kim H, Al-Shahi Salman R, McCulloch CE, Stapf C, Young WL, MARS Coinvestigators (2014). Untreated brain arteriovenous malformation: patient-level meta-analysis of hemorrhage predictors. Neurology.

[25] Kim YH, Choe SW, Chae MY, Hong S, Oh SP (2018). SMAD4 Deficiency leads to development of arteriovenous malformations in neonatal and adult mice. J Am Heart Assoc.

[26] Krithika S, Sumi S (2020) Neurovascular inflammation in the pathogenesis of brain arteriovenous malformations. J Cell Physiol, in press. 10.1002/jcp.30226 10.1002/jcp.30226 33345330

[27] Kumar YK, Mehta SB, Ramachandra M (2016). Numerical modeling of vessel geometry to measure hemodynamics parameters non-invasively in cerebral arteriovenous malformation. Biomed Mater Eng.

[28] Kumar YK, Mehta SB, Ramachandra M (2017). Computer simulation of Cerebral Arteriovenous Malformation-validation analysis of hemodynamics parameters. PeerJ.

[29] Li CQ, Hsiao A, Hattangadi-Gluth J, Handwerker J, Farid N (2018). Early hemodynamic response assessment of stereotactic radiosurgery for a cerebral arteriovenous malformation using 4D flow MRI. AJNR Am J Neuroradiol.

[30] Ma L, Guo Y, Zhao YL, Su H (2015). The role of macrophage in the pathogenesis of brain arteriovenous malformation. Int J Hematol Res.

[31] MacDonald ME, Dolati P, Mitha AP, Wong JH, Frayne R (2016). Flow and pressure measurements in aneurysms and arteriovenous malformations with phase contrast MR imaging. Magn Reson Imaging.

[32] McCormick WF (1966). The pathology of vascular (“arteriovenous”) malformations. J Neurosurg.

[33] Mouchtouris N, Jabbour PM, Starke RM, Hasan DM, Zanaty M, Theofanis T, Ding D, Tjoumakaris SI, Dumont AS, Ghobrial GM, Kung D, Rosenwasser RH, Chalouhi N (2015). Biology of cerebral arteriovenous malformations with a focus on inflammation. J Cereb Blood Flow Metab.

[34] Mullan S (1994). Reflections upon the nature and management of intracranial and intraspinal vascular malformations and fistulae. J Neurosurg.

[35] Neyazi B, Herz A, Stein KP, Gawish I, Hartmann C, Wilkens L, Erguen S, Dumitru CA, Sandalcioglu IE (2017). Brain arteriovenous malformations: implications of CEACAM1-positive inflammatory cells and sex on hemorrhage. Neurosurg Rev.

[36] Nikolaev SI, Vetiska S, Bonilla X, Boudreau E, Jauhiainen S, Rezai Jahromi B, Khyzha N, DiStefano PV, Suutarinen S, Kiehl TR, Mendes Pereira V, Herman AM, Krings T, Andrade-Barazarte H, Tung T, Valiante T, Zadeh G, Tymianski M, Rauramaa T, Ylä-Herttuala S, Wythe JD, Antonarakis SE, Frösen J, Fish JE, Radovanovic I (2018). Somatic activating KRAS mutations in arteriovenous malformations of the brain. N Engl J Med.

[37] Nishida T, Faughnan ME, Krings T, Chakinala M, Gossage JR, Young WL, Kim H, Pourmohamad T, Henderson KJ, Schrum SD, James M, Quinnine N, Bharatha A, Terbrugge KG, White RI (2012). Brain arteriovenous malformations associated with hereditary hemorrhagic telangiectasia: gene-phenotype correlations. Am J Med Genet A.

[38] Ognard J, Magro E, Caroff J, Ben Salem D, Andouard S, Nonent M, Gentric JC (2018). A new time-resolved 3D angiographic technique (4D DSA): Description, and assessment of its reliability in Spetzler-Martin grading of cerebral arteriovenous malformations. J Neuroradiol.

[39] Orlowski P, Mahmud I, Kamran M, Summers P, Noble A, Ventikos Y, Byrne JV (2014). An approach to the symbolic representation of brain arteriovenous malformations for management and treatment planning. Neuroradiology.

[40] Park SO, Lee YJ, Seki T, Hong KH, Fliess N, Jiang Z, Park A, Wu X, Kaartinen V, Roman BL, Oh SP (2008). ALK5- and TGFBR2-independent role of ALK1 in the pathogenesis of hereditary hemorrhagic telangiectasia type 2. Blood.

[41] Pawlikowska L, Tran MN, Achrol AS, McCulloch CE, Ha C, Lind DL, Hashimoto T, Zaroff J, Lawton MT, Marchuk DA, Kwok PY, Young WL, UCSF BAVM Study Project (2004). Polymorphisms in genes involved in inflammatory and angiogenic pathways and the risk of hemorrhagic presentation of brain arteriovenous malformations. Stroke.

[42] Petridis AK, Dibue-Adjei M, Cornelius JF, Suresh MP, Li L, Kamp MA, Abusabha Y, Turowski B, Steiger HJ, May R (2018). Contrast enhancement of vascular walls of intracranial high flow malformations in black blood MRI indicates high inflammatory activity. Chin Neurosurg J.

[43] Priemer DS, Vortmeyer AO, Zhang S, Chang HY, Curless KL, Cheng L (2019). Activating KRAS mutations in arteriovenous malformations of the brain: frequency and clinicopathologic correlation. Hum Pathol.

[44] Raoult H, Bannier E, Maurel P, Neyton C, Ferré JC, Schmitt P, Barillot C, Gauvrit JY (2014). Hemodynamic quantification in brain arteriovenous malformations with time-resolved spin-labeled magnetic resonance angiography. Stroke.

[45] Ruedinger KL, Harvey EC, Schafer S, Speidel MA, Strother CM (2019). Optimizing the quality of 4D-DSA temporal information. AJNR Am J Neuroradiol.

[46] Sandoval-Garcia C, Yang P, Schubert T, Schafer S, Hetzel S, Ahmed A, Strother C (2017). Comparison of the diagnostic utility of 4D-DSA with conventional 2D- and 3D-DSA in the diagnosis of cerebrovascular abnormalities. AJNR Am J Neuroradiol.

[47] Scianna M, Bell CG, Preziosi L (2013). A review of mathematical models for the formation of vascular networks. J Theor Biol.

[48] Sekhar LN, Wasserman JF (1984). Noninvasive detection of intracranial vascular lesions using an electronic stethoscope. J Neurosurg.

[49] Sorimachi T, Takeuchi S, Koike T, Minakawa T, Abe H, Tanaka R (1995) Blood pressure monitoring in feeding arteries of cerebral arteriovenous malformations during embolization: a preventive role in hemodynamic complications. Neurosurgery 37(6):1041-7. discussion 1047-8. 10.1227/00006123-199512000-00002 10.1227/00006123-199512000-00002 8584143

[50] Steiger HJ, Poll A, Liepsch D, Reulen HJ (1987). Basic flow structure in saccular aneurysms: a flow visualization study. Heart Vessels.

[51] Thompson DW (1942) On growth and form, 2nd edn. Cambridge University Press, Cambridge

[52] Walcott BP, Winkler EA, Zhou S, Birk H, Guo D, Koch MJ, Stapleton CJ, Spiegelman D, Dionne-Laporte A, Dion PA, Kahle KT, Rouleau GA, Lawton MT (2018). Identification of a rare BMP pathway mutation in a non-syndromic human brain arteriovenous malformation via exome sequencing. Hum Genome Var.

[53] Winkler EA, Lu AY, Raygor KP, Linzey JR, Jonzzon S, Lien BV, Rutledge WC, Abla AA (2019). Defective vascular signaling & prospective therapeutic targets in brain arteriovenous malformations. Neurochem Int.

[54] Wright R, Järvelin P, Pekonen H, Keränen S, Rauramaa T, Frösen J (2020). Histopathology of brain AVMs part II: inflammation in arteriovenous malformationof the brain. ActaNeuro chir (Wien).

[55] Zhang R, Han Z, Degos V, Shen F, Choi EJ, Sun Z, Kang S, Wong M, Zhu W, Zhan L, Arthur HM, Oh SP, Faughnan ME, Su H (2016). Persistent infiltration and pro-inflammatory differentiation of monocytes cause unresolved inflammation in brain arteriovenous malformation. Angiogenesis.

[56] Zhu W, Shen F, Mao L, Zhan L, Kang S, Sun Z, Nelson J, Zhang R, Zou D, McDougall CM, Lawton MT, Vu TH, Wu Z, Scaria A, Colosi P, Forsayeth J, Su H (2017). Soluble FLT1 gene therapy alleviates brain arteriovenous malformation severity. Stroke.

[57] Zhu W, Chen W, Zou D, Wang L, Bao C, Zhan L, Saw D, Wang S, Winkler E, Li Z, Zhang M, Shen F, Shaligram S, Lawton M, Su H (2018). Thalidomide reduces hemorrhage of brain arteriovenous malformations in a mouse model. Stroke.

